# The New Year and Medical Journals

**Published:** 1858-01

**Authors:** 


*The New Year and Medical Journals.*—The December numbers of our exchanges have generally contained some reference to their prospects and prosperity, appropriate to the close of a volume. Many of them have been conducted through quite a number of years and may be said to have at least arrived at years of maturity if not of discretion; the little paper which we welcome every week from Boston, has now arrived at an advanced age. The Western Lancet is also antiquated, having numbered as many as eighteen summers. The Southern Journal, The Nashville Journal, and The Medical News & Library, are our seniors, as well as the American and the New New York Journals. Of all the periodicals on our list of exchanges, The American Journal is the oldest, having been issued in its new series, for twenty-two years. The North American Medico Chirurgical Review is the youngest, having just finished its first volume. This periodical is the result of a fusion of The Louisville Review, which took the place of The Western Journal, with The Medical Examiner. We see by the last number that it is about to be enlarged, and to present yearly abstracts of the American contributions to the various departments of medical science.

We are glad to see so many contributions to the periodical literature of the profession, as there have been during the past year, and we think that we have good reason to congratulate ourselves on the able list of colabora-tors to which our pages have been so much indebted for their interest, and which has made the task of the editors comparatively light to what it has been in years gone by. With such an array of names as we have, as regular contributors, it is impossible that this should be anything but a good Journal, even though the editor used no endeavors to render it such; very much as many diseases will pass to a favorable termination without the aid of the doctor and sometimes even in spite of him. Were our subscribers as liberal as our contributors, this would also become one of the best paying journals of its class in the country. F.

